# Whey Protein Isolate and β-Lactoglobulin-Modified Alginate Hydrogel Scaffolds Enhance Cell Proliferation for Cultivated Meat Applications

**DOI:** 10.3390/foods14142534

**Published:** 2025-07-19

**Authors:** Irfan Tahir, Christopher Foley, Rachael Floreani

**Affiliations:** 1 Department of Mechanical Engineering, University of Vermont, Burlington, VT 05401, USA; itahir@uvm.edu (I.T.); christopher.foley@uvm.edu (C.F.); 2 Department of Electrical and Biomedical Engineering, University of Vermont, Burlington, VT 05401, USA; 3 Materials Science Program, University of Vermont, Burlington, VT 05401, USA; 4 Food Systems Program, University of Vermont, Burlington, VT 05401, USA

**Keywords:** whey protein, β-lactoglobulin, cultivated meat, scaffold, muscle cells

## Abstract

Innovative changes to our current food system are needed, and one solution is cultivated meat, which uses modern engineering, materials science, and biotechnology to produce animal protein. This article highlights the advantages of incorporating whey protein isolate (WPI) and β-lactoglobulin (β-LG) into hydrogel networks to aid cell growth on cultivated meat scaffolds. The protein and polysaccharide (i.e., alginate) components of the scaffolds are food-grade and generally regarded as safe ingredients, enabling the transition to more food-safe, edible, and nutritious scaffolds. The impact of WPI and varying properties on cell performance was evaluated; alginate concentration and the addition of proteins into the hydrogels significantly altered their stiffness and strength. The results of this study demonstrate the innocuous nature of novel scaffolds and reveal enhanced cell proliferation on WPI and β-LG-modified groups compared to standard biomaterial controls. This work serves as a stepping stone for more comprehensive analyses of WPI, β-LG, and alginate scaffolds for use in cultivated meat research and production.

## 1. Introduction

Industrial animal agriculture is under pressure. As the appetite for meat increases, cultivated meat may provide a solution to relieve the pressure on our current food system [1,2]. Cultivated meat production includes four stages: (1) collection of cells from an animal, (2) seeding cells on a substrate, (3) proliferating cells in a bioreactor, and (4) packaging the final product [3,4,5]. Remaining technical barriers hinder cultivated meat’s commercial viability; one challenge is engineering material scaffolds to recapitulate the necessary structural, mechanical, and biochemical cues cells need to effectively expand, differentiate, and form tissue [6,7]. Indirect physical signals (e.g., density, stiffness, and surface charge) from the cell substrate to adherent cells affect the behavior, metabolism, and phenotype of cells; this process is known as mechanotransduction, and it is widely accepted as a valuable tool in tissue engineering [8,9,10]. Appreciating and understanding mechanotransduction at even the basic level for cultivated meat is critical not only for tissue development but as it relates to the texture and taste of the meat product. Designing the optimal scaffold involves selecting parameters and property targets, such as component selection, crosslinking, and tuning of mechanical properties, which present challenges in accurately portraying muscle tissue’s structural and biochemical properties to guide proper tissue development [6,7,10]. One common material used for scaffolding is sodium alginate.

Alginate is an inexpensive, highly abundant polysaccharide derived from brown algae amendable to modification with high workability; it can form hydrogels in physiological conditions [11,12]. Despite these advantages, alginate lacks cell adhesive sites to enable cell proliferation and tissue growth [13]. To encourage cell adhesion, alginate is commonly surface-modified with the cell adhesion ligand arginyl–glycyl–aspartic acid (RGD) [14,15,16,17,18]. Alginate hydrogels are recognized for their mechanical properties, which can be fine-tuned by changing their molecular weight, chemical composition (repeat unit modification versus copolymer formulation), and crosslinking methodology. Divalent cations, such as calcium (Ca^2+^), create a hydrogel network by iconically crosslinking adjacent carboxyl groups along the alginate’s backbone. Alginate is also amendable to a variety of chemistries to design a material with specific functionalities [19,20]. The elastic modulus of alginate can be fine-tuned to resemble animal skeletal muscle tissue, which has an average elastic modulus of 10 kPa [21].

Whey permeates (i.e., whey protein) are a byproduct of the dairy industry and a valuable source of protein that have been underutilized in materials engineering. Compared to soy protein [22], pea protein [23], zein protein, and others, whey protein offers nutritional and mechanical benefits unlike what has been presented in the literature. Whey is one of two main protein complexes found in the milk of mammals and the content remaining after the removal of casein [24]. Whey permeates are refined and sold as either whey protein concentrate (WPC, 30–80% protein) or whey protein isolate (WPI, 80–94% protein) [24]. Bovine whey permeate consists of a variety of proteins, including α- and β-lactoglobulin (β-LG), lactalbumin, immunoglobulins, bovine serum albumin, bovine lactoferrin, and lactoperoxidase [24,25,26]. β-LG is the most dominant whey protein, comprising nearly 60% by weight [26]. Whey protein is often purchased as a food additive, i.e., as an emulsifying agent or source of protein for wellness and sports nutrition [27]. Other applications include drug delivery, soil conditioning, and bioplastic alternatives [28,29,30]. In its non-modified form, whey protein can self-gel when denatured or crosslink in the presence of transglutaminase [28,29], although the material and mechanical properties are less than desirable alone for tissue engineering [28,31]. Electrostatic blends of WPI with alginate and dextran, among others, form coacervates or emulgels for use in the food and medical industries, but the tunability is limited and macro-structural integrity is inadequate for supporting tissue growth [32,33,34,35,36,37,38,39,40].

Only recently has whey protein been investigated to support cell adhesion and proliferation [41,42,43,44]. WPI alone [45,46] and blends were recently shown to support mineralized bone tissue development when mixed with hydroxyapatite particles [45,46,47,48,49]. Whey protein hydrogels, however, have not been widely investigated as a substrate for muscle tissue engineering, and only limited research has been performed using whey-protein-based hydrogel for cultivated meat applications [15,50,51]. A large gap in knowledge also exists for the use of β-LG in tissue engineering. For the first time, β-LG-modified alginate scaffolds will be compared to scaffolds modified with WPI. There is a lack of evidence that β-LG has been investigated for cell adhesion throughout a hydrogel material. Therefore, the objective of this study was to fabricate and characterize WPI and β-LG-modified alginate scaffolds. Various experiments were performed to determine the viability and metabolic activity of primary bovine skeletal muscle cells (pBMCs) cultured on the scaffolds in static conditions.

## 2. Materials and Methods

### 2.1. Materials

Sodium alginate (viscosity average molecular weight = 170–240 kDa) was generously donated by FMC Biopolymer. WPI 895 (protein 94%, and total solids 95.6%) was purchased from Fonterra Ltd. Phosphate buffered saline (PBS), calcium chloride (CaCl_2_), N-hydroxysuccinimide (NHS), 1-ethyl-3-(3-dimethylaminopropyl) carbodiimide (EDC), hydrochloric acid (HCl), gelatin from bovine skin (Type B), β-LG from bovine milk, Corning Costar ultra-low attachment multiple well plates, and Triton X-100 were purchased from Sigma-Aldrich. pBMCs were isolated from a small muscle excision (~1.0 cm^3^) from the semitendinosus of a seven-month-old Simmental bull raised at the Tufts Cummings School of Veterinary Medicine (Medford, Massachusetts, USA) using approved protocols (IACUC protocol #G2018-36) and generously donated by the Kaplan Laboratory at Tufts University. Cysteine–L-arginyl–glycyl–L-aspartic acid (cRGD) was purchased from Genscript. WST-8 cell proliferation assay kits were purchased from Abnova. Deuterium oxide (D_2_O), a LIVE/DEAD Assay Kit, Trypan Blue, Alexa Fluor 594 phalloidin, Alexa Fluor 488 goat anti-rabbit IgG (H+L), Dulbecco’s modified Eagle medium (DMEM) (high-glucose, with L-Glutamine and without sodium pyruvate), trypsin–ethylenediaminetetraacetic acid (EDTA), penicillin–streptomycin (pen–strep), and fetal bovine serum (FBS) were purchased from Thermo Fisher Scientific. 4′,6-diamidino-2-phenylindole, dihydrochloride (DAPI) fluoromount mounting medium was purchased from SouthernBiotech. Dialysis tubing (molecular weight cutoff = 6–8 kDa) was purchased from Spectrum Chemical.

### 2.2. Alginate–RGD Synthesis

Sodium alginate was dissolved in deionized (DI) water (1%, *w*/*v*) at room temperature. The pH of the alginate solution was adjusted to 5.0 with HCl, and then EDC was added. After mixing for 30 min at room temperature, NHS was added. The COOH:EDC:NHS molar ratio remained consistent (1:8:3.2) for each carbodiimide reaction, wherein COOH refers to the moles of alginate carboxyl groups [52,53]. cRGD was thawed to room temperature and used as is from the supplier; the ligand was added to the functionalized alginate solution. The carbodiimide reaction was conducted for five hours at room temperature. The product, alginate–RGD, was dialyzed against DI water for three days to remove excess EDC and NHS, frozen at −80 °C and lyophilized to obtain a dry powder, and then stored at −20 °C until use.

### 2.3. Scaffold Fabrication and Characterization

Scaffold precursor solutions were prepared in DI at alginate concentrations of 1.5% (*w*/*v*) for compliant groups (i.e., C) and 3% (*w*/*v*) for stiff (i.e., S) groups to form two overall hydrogel categories (see Table 1). In addition, the experimental groups contained either β-LG or WPI (W-S, W-C, B-S, B-C), which were added at a concentration of 10% (*w*/*v*). Alginate blended with 10% (*w*/*v*) gelatin and alginate–RGD were used as positive controls (G-S, G-C, R-S, R-C). Alginate without any proteins or modifications was used as a negative control (A-S, A-C). After mixing, the respective solutions were added dropwise into custom 3D printed molds [15] of assorted sizes, depending on the application and test. The scaffold solutions were frozen at −20 °C for 20 min and then crosslinked using a 1.0 M CaCl_2_ bath for 30 min [15]. Crosslinked scaffolds were then rinsed in DI water.

Directly after ionic crosslinking, samples from each scaffold group were flash frozen in liquid nitrogen, cryo-fractured, lyophilized, and sputter-coated (Ted Pella 108 Manual Sputter Coater, Redding, California, USA) with 10 nm of gold prior to imaging using a Cressington 108 sputter coater. Scanning electron micrographs on scaffold cross-sections were collected at 50 and 150× magnification (Zeiss Sigma 300 VP Field-Emission SEM, Oberkochen, Baden-Württemberg, Germany). Images were used to qualitatively characterize the internal pore structure of the crosslinked scaffolds and make comparisons between hydrogel preparation methods and composition.

### 2.4. Equilibrium Swell Ratio and Weight Loss

The equilibrium swell ratio and weight loss of each scaffold group were quantified to analyze the material properties, following an adapted standard (ASTM D2765-11) [54]. Data were also used to qualitatively assess the physical integrity of the material and potential loss of material from the hydrogel. Directly after fabrication, samples from each scaffold group were flash frozen in liquid nitrogen and lyophilized. Initial weight measurements of dry samples (W_i_) were recorded. Next, the samples were immersed in 1 mL of PBS and then placed in a 37 °C shaker incubator at 150 rpm. After 24 h, the samples were removed from the PBS, and wet weights (W_w_) were recorded. The samples were frozen at −80 °C for 20 min and freeze-dried using a lyophilizer (Labconco FreeZone 6L, Kansas City, Missouri, USA), and a final dry mass was recorded for each sample (W_f_). The equilibrium swell ratio and weight loss were calculated for each sample as follows: swell ratio (%) = (W_w_ − W_i_)/W_i_ × 100; weight loss (%) = (W_i_ − W_f_)/Wi × 100. Samples were soaked for an additional six days, and qualitative evaluation of all the scaffold groups was also performed to assess the visual hydrolytic degradation of the materials.

### 2.5. Unconfined Compression Testing

All hydrogel scaffold groups were evaluated under uniaxial unconfined compression in the hydrated state directly after fabrication and crosslinking. Tests were performed at room temperature on a DHR-2 rheometer (TA Instruments, New Castle, Delaware, USA) equipped with a Peltier plate and steel plate geometry (8-mm Ø). Samples (h < ½Ø) were loaded onto the Peltier plate, and the geometry was lowered onto the sample to a pre-load of 0.01–0.03 N. The gap height was recorded as the original gauge length for displacement measurements. Samples were subjected to a compressive load at a deformation rate of 10 μm/second up to 50% compressive strain. Data were analyzed using analytical software (TA Instruments TRIOS, software v5.1.1.46572) and plotted using MATLAB (software version R2023b, MathWorks). The compressive moduli and strengths at 15% strain for each group were calculated from a stress–strain curve generated from force displacement data within the linear region (5–15% compressive strain) of the curve.

### 2.6. Primary Bovine Muscle Cell (pBMC) Culture and Characterization

#### 2.6.1. Cell Culture and Seeding on Scaffolds

pBMCs were thawed at passage two and cultured at 5,000 cells/cm^2^ in basal cell culture media (growth media) containing DMEM (high-glucose, with L-Glutamine and without sodium pyruvate), 10% (*v*/*v*) FBS, and 1% (*v*/*v*) pen–strep. Cells were cultured in a tissue culture incubator at 37 °C and 5% (*v*/*v*) CO_2_. Acellular scaffold precursor solutions were added dropwise into ultra-low adhesion cell culture plates, frozen at −20 °C for 20 min, and then ionically crosslinked using CaCl_2_. After crosslinking, the scaffolds were washed twice with PBS and sterilized using ultraviolet light for 20 min. Next, 50,000 cells/cm^2^ were added dropwise to the surface center of each scaffold and then placed into the incubator. After 20 min, additional growth media were added to all scaffold groups and then incubated until analysis for each time point.

#### 2.6.2. Cell Imaging

Fluorescent images were obtained to characterize the density of adhered cells on the scaffolds’ surfaces at different time points. After one and three days, growth media were removed, and the cell-seeded scaffolds were washed thrice with PBS. LIVE/DEAD fluorescence dye was added to the wells containing cells and incubated at room temperature for 15 min. The scaffolds were imaged with the fluorescence channels for LIVE (494 nm excitation/517 nm emission) and DEAD (528 nm excitation/617 nm emission) cells using a Biotek Cytation 5 microscope (Agilent Technologies, Santa Clara, California, USA) at 4× magnification.

#### 2.6.3. Cell Metabolic Activity

pBMCs were seeded at a density of 50,000 cells/cm^2^ on scaffolds placed in ultralow adhesion well plates and on standard tissue culture polystyrene (T, positive control). The metabolic activity of pBMCs cultured on scaffolds was determined on days one and three using a WST-8 Cell Proliferation Assay Kit [55,56,57,58]. Briefly, after rinsing with PBS, pBMCs cultured on scaffolds and T were incubated in 10% (*v*/*v*) WST-8 reagent in growth media for 60 min at 37 °C with 5% CO_2_. Following incubation, 100 μL aliquots of the WST-8 and growth media were transferred to 96-well plates, and the absorbance was measured at 460 nm using a microplate reader (Agilent Technologies Biotek Cytation 5, Santa Clara, California, USA).

### 2.7. Statistics

The mean and standard deviation for each sample group in the physico-mechanical analysis (weight loss, swell ratio, and unconfined compression testing) and quantitative cell assay were calculated. All experiments were performed in triplicate, unless otherwise stated. A two-way analysis of variance (ANOVA) was performed to determine the statistical significance between sample groups (* *p* ≤ 0.05, ** *p* ≤ 0.01, *** *p* ≤ 0.001). Additional post hoc Bonferroni multiple comparisons tests were performed to support significant findings.

## 3. Results

### 3.1. Scaffold Fabrication and Characterization

**Figure 1:**
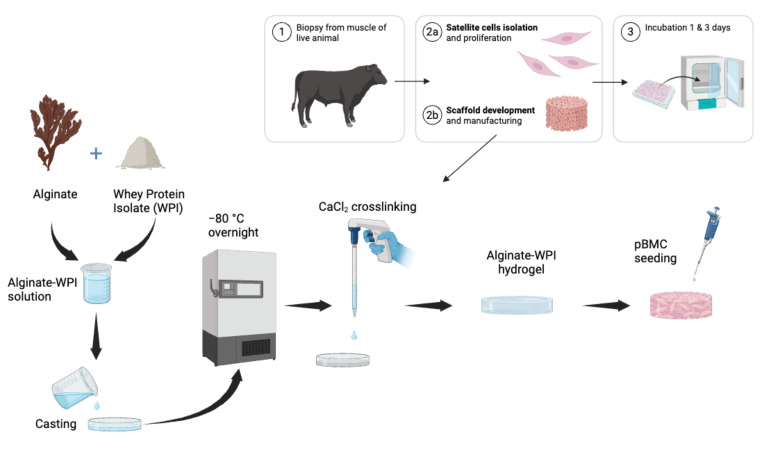
The fabrication of whey-based scaffolds was developed for applications in cultivated meat and the use of primary bovine cells. Schematic representations of alginate–WPI hydrogel scaffolds were fabricated, along with control materials, and characterized using physico-mechanical properties and biological tests.

**Figure 2:**
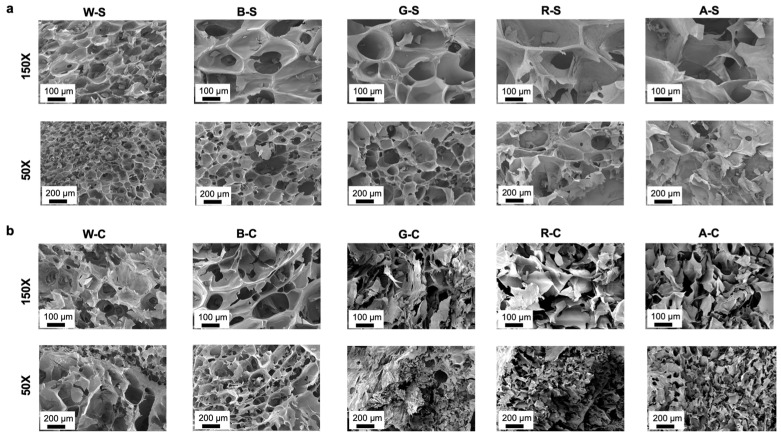
SEM images of WPI, β-LG, gelatin, RGD, and non-modified alginate scaffolds after lyophilization. Structural differences between the (**a**) stiff and (**b**) compliant experimental and control groups are shown at 50× and 150× magnification. All materials demonstrated a porous structure, with notable differences in the size and homogeneity of the pores between groups.

To assess and compare the effectiveness of whey-protein-modified alginate hydrogels for use as tissue engineering scaffolds, two cell-culture-relevant positive controls were made. Primary cells were obtained from living animals for relevance to the intended application. This study focused on applying WPI and β-LG materials for growing primary cells for use as cultivated meat scaffolds (Figure 1). Sodium alginate was chemically modified with the cell adhesion ligand RGD using previously published methods [14,15,18], and alginate was blended with gelatin as an additional control. Bulk hydrogel scaffolds were formed via ionic crosslinking of the alginate component of the hydrogels (both experimental and control groups). As pore size, porosity, and pore structure can significantly influence cell behavior, SEM was used to characterize the scaffold microstructure and qualitatively assess the porosity of the crosslinked scaffolds in their dehydrated state. The stiff hydrogel groups (see Figure 2a) consisting of WPI, β-LG, and gelatin (W-S, B-S, and G-S), had much more defined and homogenous cross-sectional porous structures. Less defined structures were observed for the RGD-modified and non-modified alginate hydrogels (R-S and A-S). Extending this observation to the compliant groups (see Figure 2b), only B-C exhibited a neatly defined porous structure (like the stiff group), whereas all other groups exhibited less homogenous and collapsed pores. The compliant groups (R-C and A-C) appeared denser, with less defined pores.

### 3.2. Equilibrium Swell Ratio and Weight Loss

**Figure 3:**
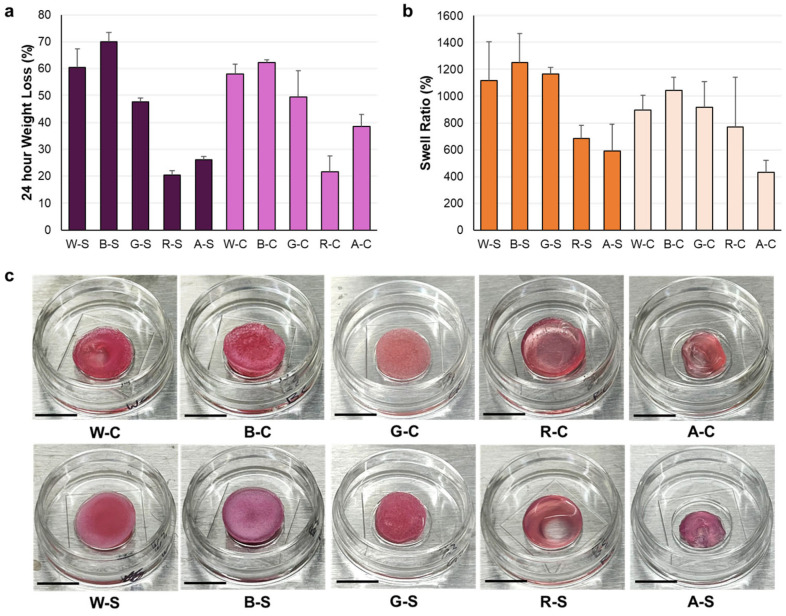
(**a**) Weight loss and (**b**) equilibrium swell ratio percentages for hydrogel scaffolds after 24 h in PBS at 37 °C. Stiff groups are shown in the dark purple and orange, while compliant groups are shown in the light purple and light orange, respectively. (**c**) Scaffold images after seven days in basal cell culture media.

The weight loss and swell ratios were measured for scaffold groups modified with proteins (W-S, W-C, B-S, B-C, G-S, and G-C) and compared to groups with protein conjugation (R-S and R-C) (Figure 3a); the R-S (20 ± 2%) and R-C (22 ± 6%) controls showed the least amount of weight loss after a 24 h soak compared to all other groups. Between stiff and compliant groups, the trends for weight loss and swell ratio were the same; the weight loss and swell ratio for the protein-modified materials were both significantly higher than those of the control. The stiff groups for the protein blends (W-S, B-S, and G-S) lost more weight compared to the compliant groups, with B-S (70 ± 3%) losing the most amount of mass. The weight loss of B-C (62 ± 1%) was highest for the compliant groups but still significantly different and lower than B-S. This is noteworthy because both stiff and compliant groups had the same amount of β-LG (10% (*w*/*v*)), but, due to the difference in alginate concentration, a higher amount of material was lost with the lower alginate concentration. Similar trends were observed between stiff and compliant groups for equilibrium swell ratio data, where the B-S (1252 ± 215%) and B-C (1044 ± 96%) groups exhibited the highest amount of swelling (Figure 3b). Qualitative evaluation of the scaffold groups was performed to visualize the degradation of the materials. Scaffolds were submerged in a dynamic environment at 37 °C. Photographs were taken after seven days (Figure 3c). Visually, the samples that contained only RGD-modified alginate and non-modified alginate controls were the most degraded. The stiff samples containing the proteins maintained their structure.

### 3.3. Mechanical Properties

**Figure 4:**
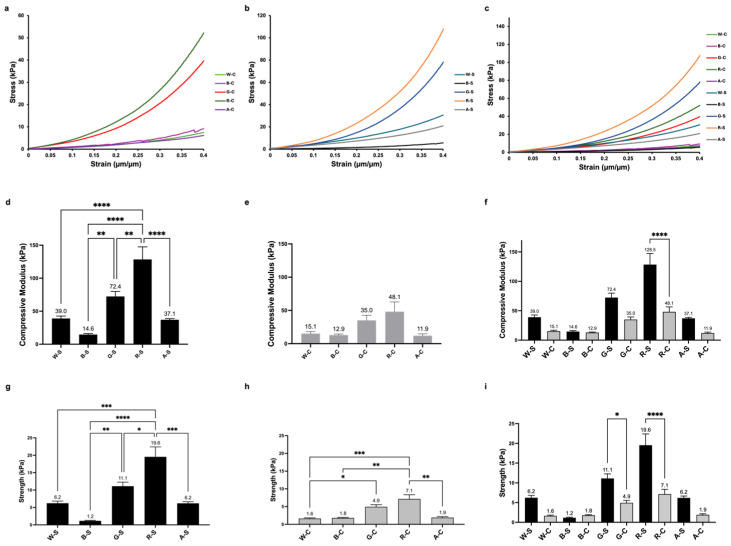
(**a**–**c**) Overlay of representative stress–strain curves comparing the mechanical response of different scaffolds under unconfined compression. (**d**–**f**) Compressive moduli and (**g**–**i**) strengths at 15% strain were calculated and organized into compliant, stiff, and combined plots. Compressive moduli data were extracted from the region of the stress–strain curves between 5 and 15% strain. Data are presented as mean ± the standard error of the mean. Significant differences are reported as * *p* ≤ 0.05, ** *p* ≤ 0.01, *** *p* ≤ 0.001, **** *p* ≤ 0.0001.

In addition to physical characterization, the scaffolds’ performance under axial compression was evaluated. The slope of the stress–strain curve in the region between 5 and 15% compressive strain only was calculated and presented as the elastic modulus, i.e., the stiffness, of the hydrogel scaffolds. The material response overall in axial compression was observed up to 40% strain. Representative stress–strain curves are shown in Figure 4a–c, highlighting the significant differences in the material response and the changes in stress with increasing strain. All samples were evaluated directly after formation in the hydrated state. Overall, there was large variation in material behavior, which was significantly dependent on the components of the material network, including the alginate concentration in the hydrogel. The compressive modulus of the RGD-modified alginate (R-S, 129 ± 19 kPa) was significantly higher than that of all of the other groups. For the stiff groups (Figure 4d), the moduli between W-S (39 ± 4 kPa), B-S (15 ± 2 kPa), and A-S (37 ± 2 kPa) were not significantly different. The only group that had significantly greater stiffness compared to W-S was R-S. Comparing the moduli between all compliant groups (Figure 4e), they were not significantly different from each other. Comparing between the compliant and stiff groups (Figure 4f), the only groups that showed a significant increase in compressive moduli with an increase in alginate concentration was the RGD-modified hydrogel group. All other groups did not significantly change their mechanical properties.

The compressive strength at 15% strain was used to compare the strength of the network structure of the WPI and β-LG-modified groups and those of the negative and positive controls. The only significant increases in compressive strength in the hydrogel scaffolds as the alginate concentration increased were the RGD and gelatin-modified groups (Figure 4i). The B-S has the lowest strength (1 ± 0 kPa), while the highest strength achieved belonged to R-S, with 20 ± 3 kPa. The W-C has the lowest strength (2 ± 0 kPa), while the highest strength achieved belonged to R-C, with 7 ± 1 kPa.

### 3.4. pBMC Bioactivity and Proliferation

**Figure 5:**
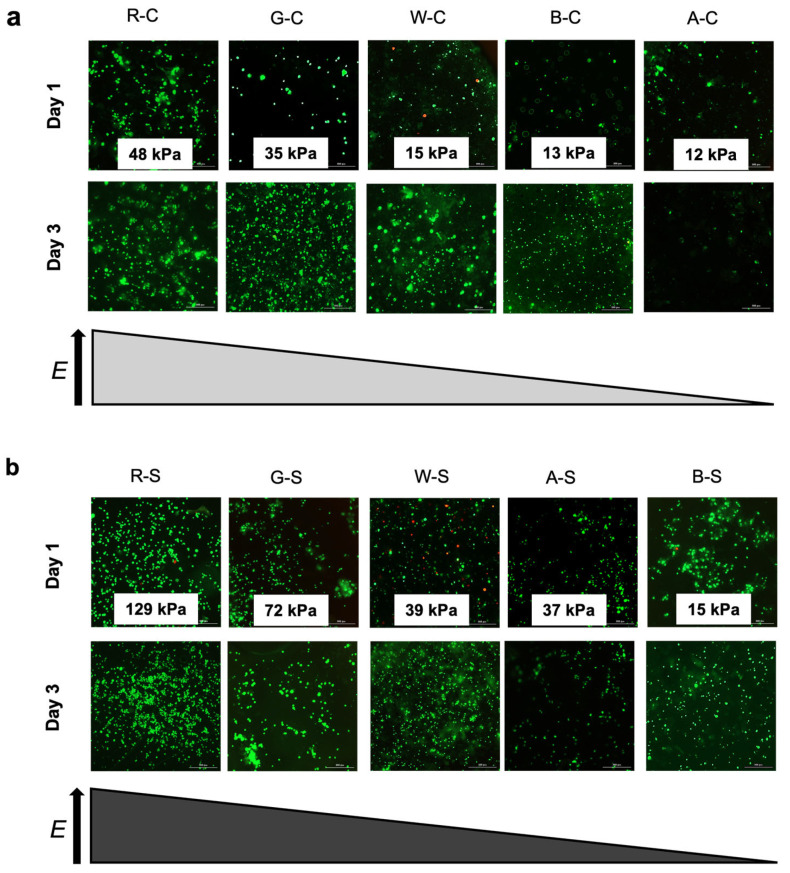
LIVE/DEAD combined images of pBMCs on (**a**) compliant and (**b**) stiff scaffold groups after one and three days of culture. Images are organized according to decreasing compressive modulus (*E*) from left to right. The compressive moduli values for each scaffold group before cell seeding are displayed on the Day 1 images. Scale bar = 300 μm.

Images of viable cells (green) on the different scaffold groups were arranged according to their compressive moduli, as shown in Figure 5a (compliant groups) and Figure 5b (stiff groups). From the 2-D fluorescent images, we see that qualitatively, the R-S group showed the greatest number of cells present on the scaffold surface over three days in culture. Both A-S and A-C images showed fewer cells from day one to day three, and those groups also showed the lowest cell density compared to the positive controls and the experimental groups. The few viable cells at day three are possibly due to growth factors, such as fibronectin and vitronectin, in growth media, as these two growth factors are known to assist with cell adhesion [17]. While the cell density appeared similar between all of the protein-modified and R groups, the whey-protein-modified scaffolds showed a large increase in cell density on the surface of the scaffold between days one and three.

**Figure 6:**
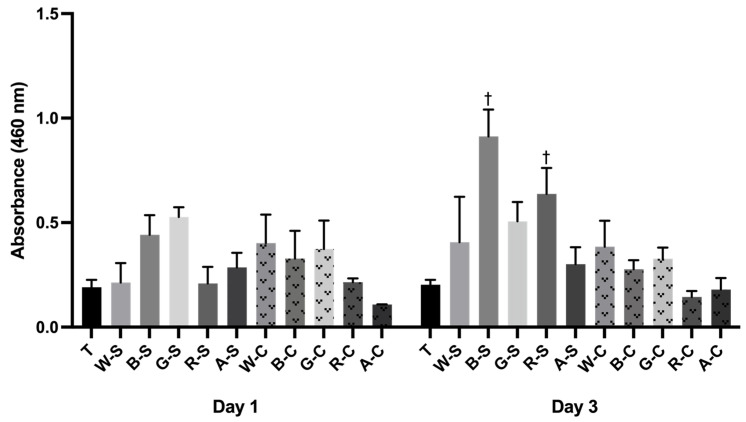
pBMCs mitochondrial activity after seeding onto scaffolds for one and three days using a WST-8 mitochondrial activity assay. Tissue culture polystyrene (T), in the absence of hydrogels, served as an assay positive control. Data are presented as mean ± the standard error of the mean. Significant differences within the same group at different time points are reported as ^†^ *p* < 0.001.

The mitochondrial activity of the cells was quantified to assess both viability and the impact of growing pBMCs on scaffolds modified with WPI and β-LG (Figure 6). Overall, the hydrogel scaffolds were not cytotoxic, which was expected based on previous research. Also, there was very little variability in mitochondrial activity after one day of culture; the only significant difference was G-S, exhibiting significantly higher (*p* ≤ 0.05) activity compared to the A-C group. After three days of culture, the B-S group supported significantly higher cell mitochondrial activity compared to all of the scaffold groups, except for R-S. B-S was slightly significantly higher than G-S (*p* ≤ 0.05) and moderately significantly higher than W-S and W-C (*p* ≤ 0.01). Out of the protein-modified groups and controls, B-S and R-S showed the only significantly increased mitochondrial activity from day one to day three (*p* ≤ 0.001), suggesting that both groups encouraged greater cell proliferation compared to the other groups.

## 4. Discussion

Herein, for the first time, β-LG was used to encourage pBMC adhesion and proliferation on cultivated meat scaffolds. The intention was to compare the new results to previous reports on WPI-modified alginate hydrogels. The data presented in this study support our hypothesis that β-LG actively contributed to the positive cell response. While purified β-LG was used, it was compared to a WPI-modified alginate hydrogel, as WPI also contains β-LG at a weight concentration of 94%. As discussed below, the addition of β-LG does indeed improve the bioactivity of alginate hydrogels; however, the physical and mechanical properties have yet to be optimized for use as cultivated meat scaffolds. The WPI-modified alginate hydrogels continue to show promise. For this study, we directly compared dairy protein hydrogels to tissue engineering standards and modified alginate hydrogels.

Scaffold development focused on creating a solid and strong hydrogel network with an appropriate structure to support cell growth for cultivated meat. To change the properties of the scaffold groups, the alginate concentration was adjusted from 1.5% to 3%, building off of our previous work [15,51]. By varying the alginate concentration of the crosslinked network, the porous structure of the scaffolds was tailorable. Ionic crosslinking was motivated by food safety and edibility, as opposed to previous studies, which utilized methacrylation chemistry [51]. While ionic crosslinking with CaCl_2_ has many benefits, it can also induce rapid crosslinking, leading to unpredictable structural variations as some parts of the alginate precursor solution gel faster than others [59]. To mitigate this disadvantage, the scaffolds were frozen and then placed in a room temperature CaCl_2_ bath. This step was optimized to slow down the gelation kinetics and increase structural homogeneity. Images of an internal structure via the x- and y-axes show an anisotropic pore organization, with a pore size range of 100–200 µm in diameter. These structures more closely resemble the pore size dimensions of decellularized skeletal muscle [60]. The ideal internal structure is an interconnected porous network for cells to migrate and that allows for nutrient transfer, with pore diameters of 5–200 µm [61]. Electron micrographs verified that modifying the composition of the scaffold influenced the internal structure (Figure 2). The R-C and A-C groups had smaller and less defined pores, indicating that the lower alginate concentration created a weak structure, which collapsed during freeze drying. Overall, the compliant groups appeared denser, which correlates with their lower swell ratios and weight losses. The stiffer materials, and specifically those modified with proteins, displayed homogeneous cross-sectional structures.

The equilibrium swell ratio and weight loss values provided insight into the integrity of the scaffold’s interpenetrating networks. Groups that included proteins blended with alginate were expected to swell the most and lose the most weight after 24 h at 37 °C in a dynamic solution due to the lower-molecular-weight species’ incorporation into the hydrogel; however, this was not the case. Visually, the samples that contained only RGD-modified alginate and non-modified alginate controls were the most degraded after seven days; quantitatively, the RGD-modified materials showed the least amount of weight loss, indicating that water activity continued past 24 h. While the WPI and β-LG-modified materials showed the most weight loss, these were minor changes over the entire seven days. Evidence of bulk erosion was minimal; however, further analysis of the structure was not performed. After the initial swelling and soaking in PBS for 24 h, it may have been possible that the electrostatic interactions between the proteins and the alginate contributed to the structural integrity of the materials.

The mechanical properties of cultivated meat scaffolds are important to consider, as they contribute to cell proliferation and provide consumers with a palatable texture. The impact of the material properties, including stiffness, on the biological response of adherent cells is well-known in the literature. The indirect physical cues from the scaffold can initiate a cell response, such as differentiation, using a process called mechanotransduction and even encourage preferred cell adhesion [8,9]. Here, the goal was to identify groups with differences in mechanical properties to consider possible effects of material stiffness on cell mechanotransduction. Because muscle cells were used in this study, it was expected that they would grow more effectively on scaffolds that exhibit mechanical properties that closely match their native environment (i.e., skeletal muscle tissue). For this study, the stiff groups were created with the intention of having a tighter molecular network due to the increase in alginate concentration; this network structure did indeed lead to increased strength and compressive moduli for both RGD and gelatin-modified groups and RGD-modified groups only, respectively. With compressive moduli ranging from 14.6 to 128.5 kPa, the stiff groups better matched native muscle tissue environments and likely contributed to their enhanced performance compared to the compliant scaffold groups, which had compressive moduli ranging from 11.9 to 48.1 kPa. Specifically, the stiffness of the WPI-modified scaffolds replicated that of skeletal muscle (~10–100 kPa) [21]. The properties can be further modified in the future to aid in the development of muscle tissue for cultivated meat. Overall, the stiffer hydrogel groups performed better, which included the RGD-modified and gelatin and WPI incorporated scaffolds. Another characteristic that may support overall cell adhesion and proliferation is the porosity and porous structure; the three groups exhibited porous structures on the SEM images. Maintaining a high amount of porosity, network integrity, and relevant mechanical properties of the hydrogels is shown as an important first step.

One goal for this study was to determine whether whey proteins, either WPI or β-LG, encouraged pBMC adhesion and proliferation at a level comparable to or better than standard alginate biomaterial cell culture controls. Due to its dominating presence, it was hypothesized that β-LG is the specific protein in WPI responsible for promoting muscle cell adhesion. For this reason, purified β-LG was chosen as an experimental bioactive component and compared to WPI. To compare the WPI and β-LG scaffolds to well-known cell adhesion ligand controls, gelatin was blended with alginate to form a hydrogel, and RGD was conjugated to the alginate backbone to make a hydrogel, respectively [16]. Alginate was used as a negative control, as it contains no cell adhesion sites. The few viable cells on the alginate negative controls at day three are possibly due to growth factors, such as fibronectin and vitronectin, in growth media, as these two growth factors are known to assist in cell adhesion [17].

Overall, the β-LG-modified hydrogel scaffolds showed the most promise moving forward for use as a cell adhesion molecule. The B-S group had a porous structure similar to skeletal muscle (100–200 µm) and released a large amount of β-LG into the aqueous media, as determined through the weight loss calculations. As β-LG is a growth factor, it is possible that the cells growing on the B-S and R-S scaffolds were metabolically more active due to the presence of soluble β-LG [62]. Indeed, a recent study determined that β-LG can be an effective FBS replacement in cell culture media [63]. Qualitative fluorescent cell images and quantitative metabolic activity support the hypothesis that whey protein, specifically β-LG, significantly increased cell adhesion and encouraged proliferation after three days of culture due to the bioactivity of the crosslinked hydrogel network and the release of soluble growth factors into the cell culture media. By fine-tuning the crosslinking density and porosity of the materials, longer time points may provide clues regarding the bioactivity of the cells, as little is known about the in vitro metabolism and the long-term effects of substrate stiffness on cell proliferation but also differentiation into the desired phenotype for different meat or seafood products.

## 5. Conclusions

This article highlights the advantages of incorporating WPI and β-LG, and the comparison between WPI and β-LG, into hydrogel networks to aid in cell adhesion and growth on cultivated meat scaffolds. The experimental protein and polysaccharide components of the scaffolds, excluding RGD, were food-grade and generally regarded as safe ingredients, enabling the transition to more food-safe and edible scaffolds. Cell study experiments demonstrated the innocuous nature of novel β-LG-alginate scaffolds and their ability to support cell adhesion and proliferation. A gap in knowledge remains regarding the effect of WPI and β-LG-modified substrates on different cell types, particularly those primary cells and cell lines being developed and studied for meat production. Future work should include further research into the structure, composition ratios, and tunable mechanical properties of β-LG and alginate scaffolds. Additionally, further research can include analysis techniques, such as Fourier transform infrared spectroscopy (FTIR), to evaluate the protein distributions on the scaffold’s surface. In summary, this study serves as a stepping stone for more comprehensive analyses of β-LG-based scaffolds for applications in cultivated meat research and production.

## Figures and Tables

**Table 1:** Hydrogel scaffold group names and the corresponding groups and compositions. Alginate concentrations were used at 1.5% and 3% (*w*/*v*), corresponding to compliant (C) and stiff (S) material groups. WPI, β-LG, and gelatin were all used at 10% (*w*/*v*).

Group Name	Grouping	Composition
W-C, W-S	Experimental	WPI + Alginate
B-C, B-S	Experimental	β-LG + Alginate
G-C, G-S	Positive Control	Gelatin + Alginate
R-C, R-S	Positive Control	RGD–Alginate
A-C, A-S	Negative Control	Alginate

## Data Availability

The original contributions presented in this study are included in the article. Further inquiries can be directed to the corresponding author.
